# T lymphocytes maintain structure and function of fibroblastic reticular cells via lymphotoxin (LT)-B

**DOI:** 10.1186/s12865-014-0033-4

**Published:** 2014-09-30

**Authors:** Lintao Zhao, Lina Liu, Jianbao Gao, Yang Yang, Chunyan Hu, Bo Guo, Bo Zhu

**Affiliations:** Institution of Cancer, Xinqiao Hospital, Third Military Mediecal University, Chongqing, 400037 China

**Keywords:** FRCs, Spleen, CCL21, CCL19, Lt-B

## Abstract

**Background:**

Although a lot is known about how Fibroblastic Reticular Cells (FRCs) can regulate T lymphocytes (T cells), little is understood about whether or how T cells can regulate FRCs.

**Results:**

This study shows that the absence of T cells inhibited the secretion of ER-TR7 by splenic FRCs, induced the structural disorder of FRCs, down-regulated the expression of the chemokine ligands CCL21 and CCL19, and weakened the homing ability of T cells to the spleen of nude mice. Transfusion of T cells from BALB/c mice restored the structure and functions of FRCs and recovered them. The expression of lymphotoxin (LT)-B was significantly downregulated in the absence of T cells from nude mice and was recovered after the transfusion of T cells. After the occlusion of the LT-B receptor, the FRCs’ structure and functions were not restored by transfusion of T cells.

**Conclusions:**

These data reveal that the absence of T cells will subject spleen FRCs to structural and functional abnormality, and weaken the homing ability of T cells to the spleen. These changes are attributed to the T-cell- derived LT-B.

## Background

The generation of immune responses requires the interaction of rare antigen-specific T lymphocytes (T cells) with dendritic cell (DC) presenting the appropriate antigen. The spontaneous interaction between them is rare in the body and only occurs in specific structures, namely the secondary lymphoid organs (SLOs) [1]. The interactions are highly dependent on their architecture [2]. SLOs contain several compartments characterized by specific resident stromal cells. The most important compartments are the B-cell and T-cell zones. The B-cell zone is composed of follicular dendritic cells (FDCs), which produce CXCL13 to attract B cells [3]. The T-cell zone (paracortex) is rich in fibroblastic reticular cells (FRCs) that express the chemokine ligands CCL19 and CCL21 to attract naive T cells and DCs [4].

FDCs are well-established players in the B-cell responses, but the importance of T-zone FRCs in adaptive immunity has been noticed only recently. FRCs can secrete abundant extracellular matrix (ECM) and form specialized conduits that transport small molecules to the T zone [5]. FRCs enwrap these conduits to form a 3-dimensional cellular scaffold that allows DCs to adhere and recirculate T cells to migrate along, thereby improving the probability of successful encounters between activated DCs and naive T cells [6].

Previous studies suggest that reduced expression of the homeostatic chemokines in lymphoid tissues will inhibit the aggregation of T cells and DCs in the T-cell zone in SLOs and thereby lower the probability of encounter between antigen-specific T cells and DCs, thus weakening the immune response intensity [7]. Besides CCL19/21, FRCs also produce interleukin (IL)-7 to promote the survival of naive T-cells [8]. Past studies focus on the effects of FRCs on T cells, but not on the effects of T cells on FRCs, which is mainly studied in the field of HIV infection. Earlier studies on HIV infection indicate that T cell absence could decrease the IL-7 secretion by FRCs, thereby further precluding the survival of T cells [9]. However, there is no report about whether T cells can affect the secretion of CCL19 and CCL21 by FRCs. Previous investigations showed that virus could spread in an uncontrolled fashion in LTb^–/–^ mice [10]; that expression of IL-7 in FRCs from LT-B knockout mice was significantly down-regulated [11]; and that LT-B is mainly expressed in T cells [12], which together suggest that the FRC-regulated T cells may also affect FRCs through secretion of factors such as lymphotoxin (LT)-B.

In this study, with a spleen model, we comprehensively analyzed the morphology, organization and function of FRCs in the absence of T cells. Our results indicate that in the absence of T cells significant changes could occur, both, in the structure of FRCs and in the secretion of CCL21/19 by FRCs, which is likely mediated through the expression of LT-B. These results suggest that T cells can play an important role in maintaining FRC function and is probably achieved through LT-B.

## Results

### The conduits of FRCs were destroyed in the absence of T cells

**Figure 1:**
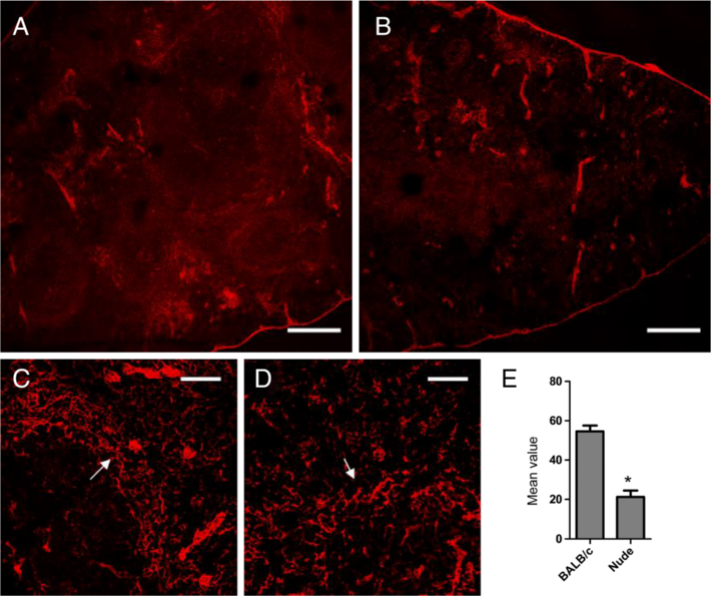
**The conduits of FRCs were destroyed in the absence of T cells.** Spleen cryostat sections from BALB/c mice **(A)** and nude mice **(B)** were stained for ER-TR7 (red = FRC-derived matrix protein), and imaged using confocal microscopy (Scale bars: 200um); Arrows show a higher magnification of the marginal zone of spleen from BALB/c mice **(C)** and nude mice **(D)** (Scale bars: 50um); We further analyzed the average fluorescence values of ER-TR7 **(E)**. Data are representative of 5 mice per group.

We first histologically studied the effects of T cell absence on splenic FRCs. FRCs form specialized conduits in the spleen and T cells move along these conduits. These conduits guide the transfer of T cells from blood to the T-cell zone [13]. ER-TR7 plays a key role in the formation of conduits and in the spleen, it is only secreted by FRCs [14]. We found that the expression of ER-TR7 was significantly downregulated in the spleens of nude mice (Figure 1A,B). We also analyzed the average values of fluorescence, the results show that ER-TR7 was significantly downregulated in the spleens of nude mice (Figure 1E); while in the spleen margins in BALB/c mice, the ER-TR7 formed a broad-banded structure which surrounded the T- and B-cell zones and was not present in the nude mice (Figure 1C,D). These findings indicate that the in the absence of T cells FRCs will undergo structural abnormality.

### T cell absence affected secretion of FRC chemokine and homing of T cells

**Figure 2:**
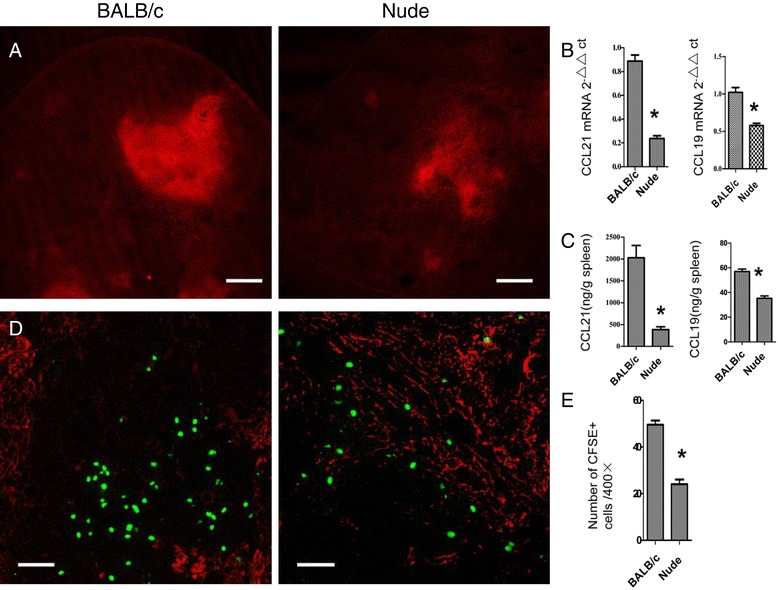
**Down-regulation of the spleen chemokines after T cell absence and altered cell localization after down-regulation of lymphoid chemokine.** Spleen cryostat sections from BALB/c mice and nude mice were stained for CCL21 (red, Scale bars: 200um), **(A)**, RT-qPCR of chemokine expression in whole spleen of BALB/c and nude mice **(B)**, chemokine expression in the spleen of BALB/c and nude mice were quantified by ELISA in tissue homogenates **(C)**, Naive T lymphocytes were purified and labeled with CFSE and transferred into mice, and their localization in the spleen white pulps was ascertained 8 hours later (Scale bars: 50um) **(D)**. Quantitation of CFSE + cells in the spleen white pulps after transfer,The number of CFSE + cells present in WP regions is shown **(E)**, Data are representative of 5 mice per group.

To further clarify whether the dysfunction of FRCs occurs following the absence of T cells, we evaluated the expressions of CCL21 and CCL19, which in the spleen were expressed only in the T-cell zone. CCL21 and CCL19 were mainly secreted by FRCs and were the key chemokines that induced the initial homing of T cells to the spleen [15]. Their RNA and protein expression levels all were significantly downregulated (Figure 2B,C). Subsequently, protein localization analysis with fluorescence microscopy also showed that the expressions of CCL21 and CCL19 were significantly downregulated (Figure 2A). We also examined the homing ability of T cells to the spleen; and, after ascertaining its specific localization to the white pulps, found that the count of CFSE-positive T cells in the splenic white pulps of nude mice was significantly reduced (Figure 2D,E).

### Transfusion of T cells recovered the splenic structure in nude mice 

**Figure 3:**
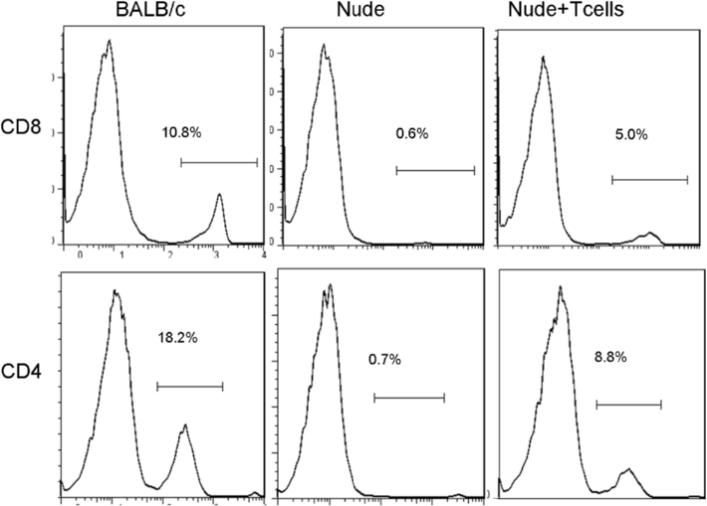
**After transfusion of T cells, the proportion of T cells in the nude mice’s spleens greatly increased.** Naive T cells were purified and transferred into nude mice every week. After four weeks, percentages of CD8^+^and CD4^+^ T cells in spleen were determined by FACS.

**Figure 4:**
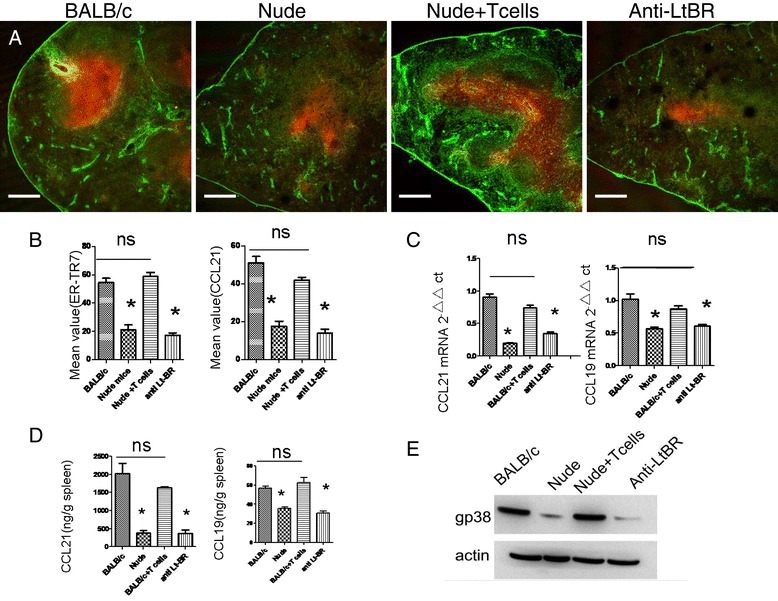
**Transfusion of T cells restored the structure of splenic FRCs and could be blocked by the antibody against the LT-B receptors.** Naive T cells were purified and transferred into nude mice every week, after four week Spleen cryostat sections from spleens of BALB/c, nude mice, nude mice + Tcells and nude mice + Tcells + anti-LtBR, mice were stained for CCL21 (red) and ER-TR7(green) (**A**, Scale bars: 200um). We further analyzed the average fluorescence values of both ER-TR7 and CCL21 **(B)**, RT-qPCR of chemokine expression in whole spleen of BALB/c, nude mice,nude mice + Tcells and nude mice + Tcells + anti-LtBR, **(C)**, chemokine expression in the spleen of BALB/c, nude mice, nude mice + T cells and nude mice + Tcells + anti-LtBR were quantified by ELISA in tissue homogenates **(D)**. Gp38 expression in whole spleen of BALB/c, nude mice, nude mice + T cells and nude mice + Tcells + anti-LtBR detected by Western blotting **(E)**. Data are representative of 5 mice per group.

**Figure 5:**
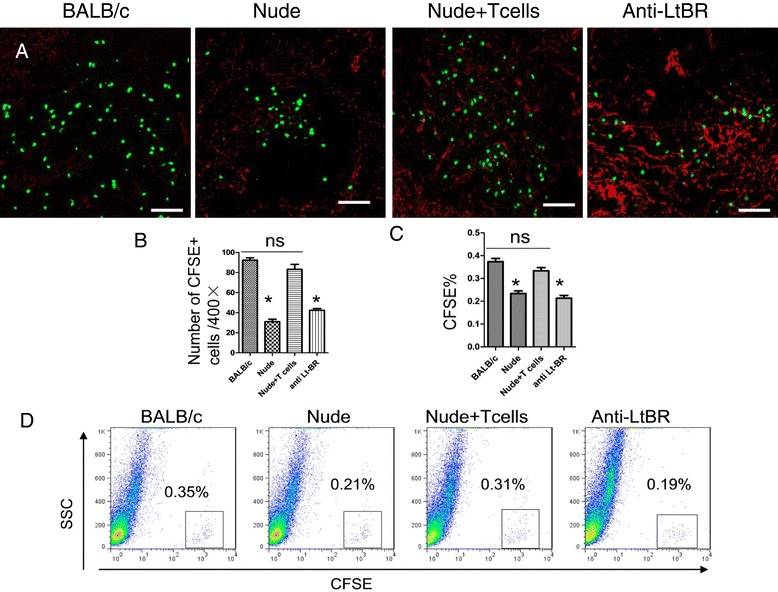
**Transfusion of T cells restored the homing-to-spleen ability of T cells and could be blocked by the antibody against the LT-B recepto**r**s.** After 4 weeks of transfusions of T cells naive T cells were purified and labeled with CFSE (green) and transferred into BALB/c, nude mice, nude mice + T cells and nude mice + T cells + anti-LtB, and their localization in the spleen was ascertained 8 hours later (Scale bars: 50um), **(A)**, Quantitation of CFSE + cells in the spleen white pulps after transfer, **(B)**, Percentages of CFSE + cells in spleen were determined by FACS, **(C,D)**. Data are representative of 5 mice per group.

We further examined whether transfusion of T cells would recover the splenic structure in nude mice. Following the transfusion of about 2-3 × 10^7^ T cells per week for 4 weeks, the proportions of CD8^+^ and CD4^+^ T-cells were greatly increased (Figure 3). Moreover, the expression of splenic ER-TR7 in nude mice was significantly improved and the structure was basically normal (Figure 4A,B). We additionally evaluated the expression of GP38, another marker of FRCs, and it also was mostly recovered (Figure 4E). The mRNA and protein expressions of CCL21 and CCL19 were also largely increased after transfusion (Figure 4A,C,D). In addition, the homing ability of T cells to the spleen white pulps was prominently enhanced (Figure 5A, B). We also assessed the proportion of CFSE-positive cells homing to the whole spleen using flow cytometry (Figure 5C,D). The results show that the transfusion of T cells improved the homing ability of T cells to the spleen.

### Participation of LT-B in structural reconstruction of FRCs

**Figure 6:**
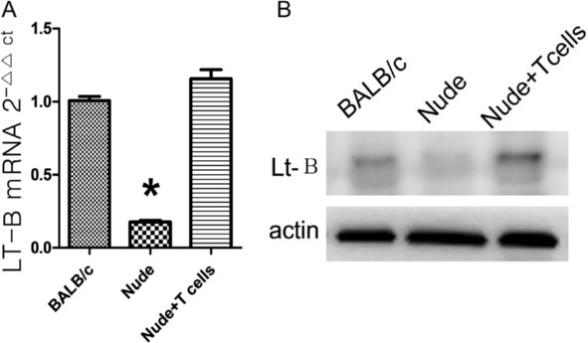
**The T cell absence induced the downregulated expression of LT-B.** Chemokine expression in whole spleen detected by RT-qPCR **(A)** and by Western blotting **(B)**, and percentages of CD8^+^ and CD4^+^ T cells determined by FACS (C) in BALB/c, nude mice and nude mice + T cells.

Previous research shows that loss of LT-B led to reduced secretion of IL-7 from FRCs. Since LT-B is mainly secreted by T cells, we further investigated the mechanism through which lymphocytes could contribute to reconstruct FRCs. We first analyzed the changes of LT-B mRNA and protein expressions and found that the expression levels in nude mice were significantly lower than in BALB/c mice (Figure 6A,B). The transfusion of T cells could upregulate the expression levels of LT-B mRNA and protein. During the transfusion, we blocked the LT-B receptor; however, the transfusion could not reconstruct the FRCs in nude mice (Figure 4A,B,E), or change the expressions of CCL21, and CCL19 (Figure 4C,D), or restore the homing ability of T cells (Figure 5).

## Discussion

In the absence of T cells splenic FRCs undergo structural and functional abnormality, and the homing ability of T cells to the spleen weakens. Transfusion of T cells can remodel the structure and functions of FRCs and promote their recovery. These changes could be attributed to the T-cell-derived LT-B. Past research shows that T cells are critical during the adaptive immune response, even though the T cells capable of identifying specific antigens are produced in the SLOs [15–17]. The stromal cells (especially FRCs) in the SLOs are critical for maintaining the structural functions of lymphoid organs and in the production of specific T cells.

Earlier research about FRCs focuses on their effects on T cells, as FRCs maintain the homeostasis of T cells via the secretion of IL-7 [8,15]. Through their secretion of CCL19 and CCL21, FRCs also chemoattract T cells to the T-cell zone in the SLOs, and thus promote the encounter between T cells and antigen presenting cells. Other studies suggest that B cells can regulate the secretion of CCL21 by FRCs, and this occurs when mice are born [18]. Research about the effects of T cells on FRCs is limited, and mainly concerns HIV infection, but it is known that in the absence of lymphocyte secretion of IL-7 by FRCs is reduced and the homeostasis of T cells weakens [add reference]. The present study showed that in the absence of lymphocyte, not only IL-7, but also the structural functions of FRCs is significantly affected.

Firstly, we found that the T cell absence led to significantly lower expression and abnormal distribution of ER-TR7. Noteworthy, FRCs in the SLOs will form conduits that are critical for regulation of T-cell-specific immune response [16,17]. These conduits are formed jointly by FRCs and the FRC-secreted ECM; especially, ER-TR7 is a key ECM and in SLOs [14], ER-TR7 is only secreted by FRCs. Such conduits rapidly transport small molecules to the T-cell zone and also guide and support the migration of initial T cells to the T-cell zone [19,20]. As the codistribution of ER-TR7 and FRCs is highly consistent, ER-TR7 is commonly used as a marker of FRCs. The expression of ER-TR7 is downregulated with the absence of T cells, especially in the marginal zone, indicating that the FRC-formed conduits dissapeared in the absence of the T cell, probably further weakening the migration and survival of T cells in the spleen.

Secondly, we found that in the absence of T cells the expressions of CCL19 and CCL21were downregulated. Interestingly, previous reports show that when lacking expression of CCL19 and CCL21 (ELC/SLC), naive T cells do not enter the T-cell zone efficiently [21,22]. Similarly, in CCR7-deficient mice, the cognate receptor for these chemokines, naive T cells cannot enter the T-cell zone, indicating the crucial role of this molecular interaction in regulating the access of naive T cell to this region. In the absence of T cell, the changes of FRC-secreted factors further weakened the homing ability of T cells. To validate this notion, we conducted the homing experiments, which yielded results that supported the above notion, as cells further damaged the homing ability of T cells and thereby weakened the immune response ability.

Finally, we found that the T cell absence significantly downregulated the LT-B level in SLOs, indicating that the reduced expressions of CCL21 and CCL19 due to T cell absence probably were induced by the absence of LT-B. These results are consistent with previous findings showing that the expression of IL-7 was significantly downregulated in the SLOs whose LT-B was knocked out and that LT-B was mainly secreted by T cells [23–25]. We also blocked the LT-B receptor and found that the transfusion of T cells did not restore the structure and functions of the spleen. This result indicated that the absence of T-cell-derived LT-B was mainly responsible for the structural and functional abnormality observed in FRCs after the T cell absence.

## Conclusions

In the absence of T cell structural and functional abnormalities appear in splenic FRCs of SLOs. The transfusion of T cells restored the structure and functions, mainly because of the T cell-derived LT-B.

## Methods

### Mice

Six-week-old female BALB/c and nude mice were purchased from the Center of Experimental Animals of Third Military Medical University (TMMU, Chongqing, China). For in vivo antibody treatment, 200 μg of mLTβR-Fc (Biogen Idec Inc., Cambridge, MA, USA) was injected at 10 d after T cell transfer, followed by 100 μg i.p. every 2 d [26]. The mice were maintained according to the TMMU Guidelines for Animal Experiments (SPF). All animal experimental protocols used in this study were in accordance with institutional Guidelines for Animal Experiments. The protocols used in the animal studies were approved by Institutional Ethics Committee of the Third Military Medical University.

### Adoptive transfers and cell migration in vivo

For in vivo cell adoptive transfer, naive splenocytes were purified using anti-CD3 magnetic beads according to the manufacturer’s instructions, and 2-3 × 10^7^ T cells were transferred into the mice every week. For in vivo cell migration, naive splenocytes were purified as above. Purified lymphocytes were labeled with 4 M carboxy fluorescein succinimidyl ester (CFSE; Molecular Probes, Eugene, OR, USA) and 5 × 10^6^ cells were transferred into each mouse [27]. After 8 hours, the mice were sacrificed and the spleens were removed for microscopic analysis. The number of CFSE + cells present in white pulp (WP) regions was assessed.

### Immunofluorescence microscopy

Spleens were removed from mice and frozen in OCT (TissueTek, Elkhart, IN, USA). The cryostat sections (10 μm) were fixed in ice-cold acetone for 10 min and then stained with the following antibodies: anti-mouse ER-TR7 (Santa Cruz Biotechnology Inc., CA, USA), and anti-mouse CCL21 (R&D Systems, Minneapolis, MN, USA) [7]. The primary antibodies were detected by fluorescein isothiocyanate (FITC)-conjugated anti-rat (1:100, Beyotime Institute of Biotechnology, Beijing, China) or Cy3-conjugated anti-goat IgG (1:200, Beyotime). Average values of fluorescence were analyzed using LAS AF Lite.

### Enzyme-linked immunosorbent assay (ELISA)

Tissues were harvested from mice and placed in phosphate buffer solution (PBS) supplemented with 1% bovine serum albumin and 1 mM pheylmethylsulfonyl fluoride (PMSF, Sigma, St Louis, MO, USA). Subsequently, tissues were homogenized. CCL21 and CCL19 were detected using a DuoSet ELISA development kit (R&DSystems) as per the manufacturer’s instructions [7].

### Western blotting

Tissues were harvested from mice and placed in RIPA buffer (Beyotime) with 1 mM PMSF (Beyotime); and then homogenized. The lysates were normalized to equal amounts of protein, and the proteins were separated by 15% gradient sodium dodecyl sulfate-polyacrylamide gel electrophoresis (SDS-PAGE), transferred to polyvinylidene fluoride (PVDF) and probed with Lt-B (Santa Cruz) or gp38 (R&D Systems). Detection was conducted by incubation with species-specific horseradish peroxidase (HRP)-conjugated secondary antibodies. Immunoreactive bands were visualized by enhanced chemiluminescence (Millipore, Billerica, MA, USA).

### Real-time quantitative reverse transcription polymerase chain reaction (RT-qPCR)

PCR primer pairs, including their specific target gene, orientation (F: forward, R: reverse), and sequence were as follows: CCL21 (F ccctggacccaaggcagt, R ggcttagagtgcttccggg), CCL19 (F ctgcctcagattatctgccat, R tcattagcaccccccagagt), actin (F cctgaggctcttttccagcc R agaggtctttacggatgtcaacgt) [7]. Whole spleens were placed in Trizol (Invitrogen, San Diego, CA, USA) and total RNA was extracted according to the manufacturer’s instructions. Real-time RT-qPCR was performed for total chemokine mRNA using SYBR Green (Takara Bio Inc., Japan) and detected using a BioRad RT-qPCR analyzer. Conditions were as follows: 95°C for 30s, then 40 cycles of 95°C for 5 s, 59°C for 34 s, then 95°C for 15 s, 60°C for 60s, and 95°C for 15 s. Expression was quantified with actin as a reference as -ΔΔCT, as per manufacturer’s instructions (Applied Biosystems).

### Statistical analysis

Statistical analysis was performed with two-tailed unpaired t-test using Prism (Graphpad Software Inc., San Diego, CA, USA). Each group contained N = 5 mice. Nude mice were randomly allocated to each group.
